# Evaluation of the cervix tissue homogeneity by ultrasound elastography in infertile women for the prediction of embryo transfer ease: a diagnostic accuracy study

**DOI:** 10.1186/s12958-017-0283-0

**Published:** 2017-08-14

**Authors:** Antonio Stanziano, Anna Maria Caringella, Clementina Cantatore, Giuseppe Trojano, Ettore Caroppo, Giuseppe D’Amato

**Affiliations:** 1 Asl Bari, DPT Maternal and Child Health, Reproductive and IVF Unit, PTA “F Jaia”, 70014 Conversano (Ba), Italy; 2 University of Bari, Obstetrics and Gynecology, 70100 BARI (Ba), Italy

**Keywords:** Elastography, Embryo transfer, Cervical canal, Female infertility, Assisted reproduction

## Abstract

**Background:**

Ultrasound elastography is a non-invasive medical imaging technique able to quantitatively characterize the stiffness of a given tissue. It has been shown to predict the risk for cervical insufficiency and preterm delivery, and to allow differentiation of malignancy from normal tissue. The present study sought to evaluate whether cervical tissue dishomogeneity, as assessed by cervical ultrasound elastography, may predict the embryo transfer (ET) ease in infertile women undergoing IVF/ICSI.

**Methods:**

We evaluated 154 infertile patients with no history of previous ET or intrauterine insemination. Cervical stiffness was evaluated in six regions of interest (ROI), compared two by two to obtain strain ratio (SR) values. Since a SR value of 1 was suggestive of tissue homogeneity, we computed 1-SR/SR-1 values to obtain a measure of the degree of cervical tissue dishomogeneity that we named “dishomogeneity index” (DI). Ultrasound-guided ET was performed by an expert operator blinded to the results of cervical elastography. The prediction ability of elastography on ET ease was evaluated by binary logistic regression, and the predictive accuracy of the independent variables was quantified with area under the curve (AUC) estimates derived from receiver operating characteristic (ROC) curve.

**Results:**

ET resulted to be easy in 99 out of 154 patients (64,2%), difficult in 54 patients (35%), and impossible in one. DI values in cervical medial lips region correctly classified 86.9% of patients, according to binary logistic regression, with a sensitivity of 81.4% and a specificity of 89,9%, positive likelihood ratio (LR) 8.07 and negative LR of 0.21. A DI cut-off value of 0.29 predicted a difficulty of ET with a sensitivity of 88,9% and a specificity of 85%.

**Conclusions:**

Cervical ultrasound elastography, by allowing the identification of cervical tissue dishomogeneity, may be of help in predicting the ET ease in infertile women candidates to IVF/ICSI.

## Background

Embryo transfer (ET) is the final and most vulnerable step in the sequential events of an in vitro fertilization (IVF) cycle. Several factors may affect ET, including initiation of uterine contractility that may provoke the immediate or delayed expulsion of the embryos, contamination of the catheter tip and inadequate ET technique [1, 2]. Cervical stenosis can be a finding in patients with difficult ET, due to underlying pathology such as post-infection or post-surgery processes.

Difficult transfers may reduce the chance of pregnancy in a IVF setting [3–5], as the use of outer catheter, stylet, and tenaculum involves a progressive reduction in clinical pregnancy rate [5]; since cervical dilatation may improve the pregnancy rate in patients with previous difficult ET [6, 7], the accurate prediction of the ET ease could allow the identification of those patients that should undergo cervical dilatation prior to the ET in order to improve their IVF outcome.

Unfortunately, the ET ease in individual patients is a largely unpredictable factor. The only diagnostic tool available to date is the mock transfer, but its reliability is questionable due to the lack of eligible studies in the field [8].

Ultrasound elastography is a non-invasive medical imaging technique that aims to quantitatively characterize the stiffness of a given tissue [9]. Recent studies suggest that it may represent a complementary method to identify women at risk for cervical insufficiency [10] or preterm delivery [11]. Elastography has been also used for the evaluation of the cervix in healthy pre-menopausal and post-menopausal women as well as in patients with cervical pathology [12]. Although the cervix was found to be characterized by a predominance of tissue of medium hardness that did not vary with age, difference in tissue stiffness as evaluated by elastography allowed differentiation of malignancy from normal tissue, in accordance with the findings of previous studies performed on other tissues.

It may be hypothesized that cervical histological changes in patients with cervical pathology could lead to difficult ET in a IVF setting. Since the above cited studies have demonstrated that cervical elastography is able to detect difference in tissue stiffness, we designed a diagnostic accuracy study aiming at evaluating whether the cervical elastography findings may predict the ease of ET in infertile women candidates to IVF. To the best of our knowledge this is the first study being sought to address this issue.

## Methods

### Study design

Data collection was planned before the tests were performed. Among all women referring to our center for couple infertility, we enrolled those that met the following entry criteria: infertility defined as failure to conceive after at least 12 months of unprotected intercourse, patients with no history of intrauterine insemination (IUI) or IVF cycles, no previous history of cervical diseases, cervical surgery or infection. All eligible patients signed the informed consent before being enrolled to the study.

One hundred fifty-four consecutive patients that fulfilled the entry criteria were admitted to IVF/ICSI cycles during the study period (February–May 2016). Blood samples were obtained from all patients to assess their basal FSH, LH, Estradiol and AMH serum levels.

Elastography was performed early in the morning on the menstrual day 2, then patients started daily hMG (Meropur® 1200 U.I., Ferring, Milano, Italy) treatment according to individualized protocols of controlled ovarian hyperstimulation. After 5 days the dosage of hMG was adjusted according to the individual ovarian response. GnRH antagonist (Orgalutran® 0,25 mg, MSD, Italy) was administered when at least one follicle measured >14 mm in diameter. Oocyte trigger was induced by administering 10.000 IU hCG (Gonasi® HP, Ibsa, Italy) 34–36 h prior to egg retrieval. The luteal phase was supported by vaginal progesterone, 200 mg three times a day (Progeffik® 200 mg Effik, Italy). Embryo transfer was performed on day 3–5 after egg retrieval by the same operator, who was blinded to the results of elastography.

### Ultrasound elastography

Real-time cervical elastography was performed by the same operator (A.S.) with more than ten years of experience in gynecologic ultrasound. The operator was trained prior to the beginning of the study by evaluating at least 100 patients and analyzing a minimum of 300 elastography images.

The cervix was evaluated using transvaginal probe (5–9 MHz) and the elastographic images were processed by the ElaXto® software on MyLab® Twice (Esaote, Genova, Italy). The vaginal probe was placed in the anterior fornix of the vagina and a sagittal view of the cervix was obtained, with a clear image of the endocervical canal, the internal and external orifice and with a similar size of anterior and posterior cervical lips. A perpendicular pressure was applied by the probe through gentle and rhythmic movements each lasting for about 1 s, exerted along the longitudinal axis of the cervix, avoiding the lateral and longitudinal dislocation of the tissues, as suggested by previous studies [13, 14]. The echo signal modification were then recorded and the tissue distortion (for soft tissues) or movements (for hard tissues) from the initial probe position were computed in real time.

In order to check the quality of the movement exerted, the transducer movement was monitored in the real-time B-Mode displayed on the left panel of the screen, while the real-time elastography was contemporarily displayed on the right panel using the split-screen mode.

**Fig. 1:**
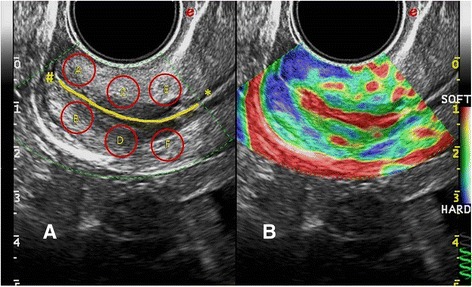
Cervical elastography. **a** Identification of six regions of interest (ROI) in the cervix. The cervical canal is highlighted in *yellow*, and the internal and external uterine orifice are indicated with the symbol * and # respectively; **b** Cervical elastography in a normal case. The difference in tissue stiffness is expressed with a *color scale*. The *green* spring on the *lower right* end side is a real-time feedback of the correct performance of the strain image acquisition

During the elastographic evaluation, the displayed color map represented the deformability degree on the area under analysis, and the waveforms of compression and decompression were displayed automatically (Fig. 1). When the pressure was applied to the cervix with the vaginal transducer, the rate of change in tissue displacement was computed, and the resulting strain value was displayed in different colors on a continuous scale ranging from red (softer tissues) to blue (stiffer tissues). A green spring was a real-time feedback of correct performance of the strain image acquisition. The procedure was then repeated in order to acquire two raw datasets for each patient.

Raw data evaluation and strain calculation were performed off-line after the end of recruitment time by MyLab-Desk® software, version: 10.0 (Esaote, Genova, Italy). In order to assess the reliability of the measurements, we repeated the off-line calculation twice for each patient within a given time interval able to render the observer blind to his first review.

We selected six opposite (as separated by the cervical canal) regions of interest (ROI) in the cervix located near to the external uterine orifice (A and B), in the median zone (C and D) and near to the internal uterine orifice (E and F) respectively (Fig. 1); each ROI was delimited by a circular area of 56,35 mm2 and their strain was calculated by the software, the resulting value being directly proportional to the stiffness of each zone under examination. The A/B, C/D and E/F strain ratio were then calculated: a value of 1 corresponded to 100% homogeneity among the two ROI under consideration and, consequently, strain ratio values different from 1 were expression of the degree of tissue dishomogeneity. In order to obtain a measure of tissue dishomogeneity we computed the difference from 1 with the formula 1-strain ratio/strain ratio-1, and the resulting values were expressed as continuous variables. We will refer to these values in the following sections by using the term dishomogeneity index (DI).

### ET procedure

Since the efficacy of the ET procedure is strictly operator-dependent [15], to avoid an experimenter bias all the ET procedures were performed by the same expert operator with more than 1000 ET performed in his career (G.D.), who was blinded to the results of cervical elastography.

After insertion of the speculum, the cervix was copiously washed with culture medium. The mucus of the internal orifice was washed with a sterile gauze soaked in saline medium and any excess was removed using a cytobrush. The Edwards Wallace catheter (Smiths medical, Hranice, Czech Republic) was first flushed with transfer medium, hyaluronan enriched (UTM®, Origio, Målov, Denmark), the total volume being 20 μL, then smoothly introduced through the cervical canal under ultrasound guidance. The embryos were injected as gently as possible while withdrawing the catheter very slightly, approximately 0.5 cm from the fundus. The catheter was then returned and inspected by the embryologist to check for retained embryos and document the absence of any blood, mucus, or endometrial tissue.

The embryo transfer was considered to be easy when it took place smoothly, without the use of the stylet or any other instrumentation, or difficult when a malleable stylet had to be used, with or without the aid of a tenaculum.

### Statistical analysis

Basal hormonal parameters in patients stratified in two groups according to the ET ease were evaluated by two-tailed Mann–Whitney U test. The prediction ability of elastography in discriminating the cases with easy and difficult transfer was evaluated by binary logistic regression, identifying the pattern of transfer (easy or difficult) as binary dependent variable, and the DI values as independent variable. The DI values obtained from the comparison of the six ROI (A/B, C/D, E/F) were separately evaluated. 95% confidence intervals were used to quantify uncertainty.

The predictive accuracy of the independent variables was quantified with area under the curve (AUC) estimates derived from receiver operating characteristic (ROC) curve.

The sample size required to estimate sensitivity was calculated according to Buderer [16]. The required samples size N should be the larger of N1 and N2 + W (maximum clinically acceptable width of the 95% CI that can be assumed =10%) computed as follows:

$$ \mathrm{N}1=\mathrm{TP}+\mathrm{FN}/\mathrm{P}\ \mathrm{N}2=\mathrm{FP}+\mathrm{TN}/\left(1\hbox{-} \mathrm{P}\right) $$

where TP = true positive, FN = false negative, FP = false positive, TN = true negative P = prevalence of the disease in the population of interest. The proportion of difficult embryo transfer was obtained by the results of a recent meta-analysis [4] which indicated that 881/2152 ET (41%) were considered to be difficult. According to the diagnostic accuracy of the strain ratio C/D as computed by the binary logistic regression, N1 (44 + 10/0.41) was 132 and N2 (10 + 89/0.59) was 167. Therefore we assumed that the required sample size N had to be at least 167 + 16.7 = 150–183 subjects.

Statistical significance was set at *p* < 0.05 for all analyses. All computations were performed using SPSS (IBM, Armonk, New York, USA) for Windows.

### IRB approval

The present prospective diagnostic study was approved by the local Independent Ethical Committee (IEC) on January 27, 2016 (IEC approval n. 4953/2016). The study have been performed in accordance with the ethical standards as laid down in the 1964 Declaration of Helsinki and its later amendments or comparable ethical standards.

## Results

All patients completed the study, and data were available for the off-line evaluation since June 2016.

**Fig. 2:**
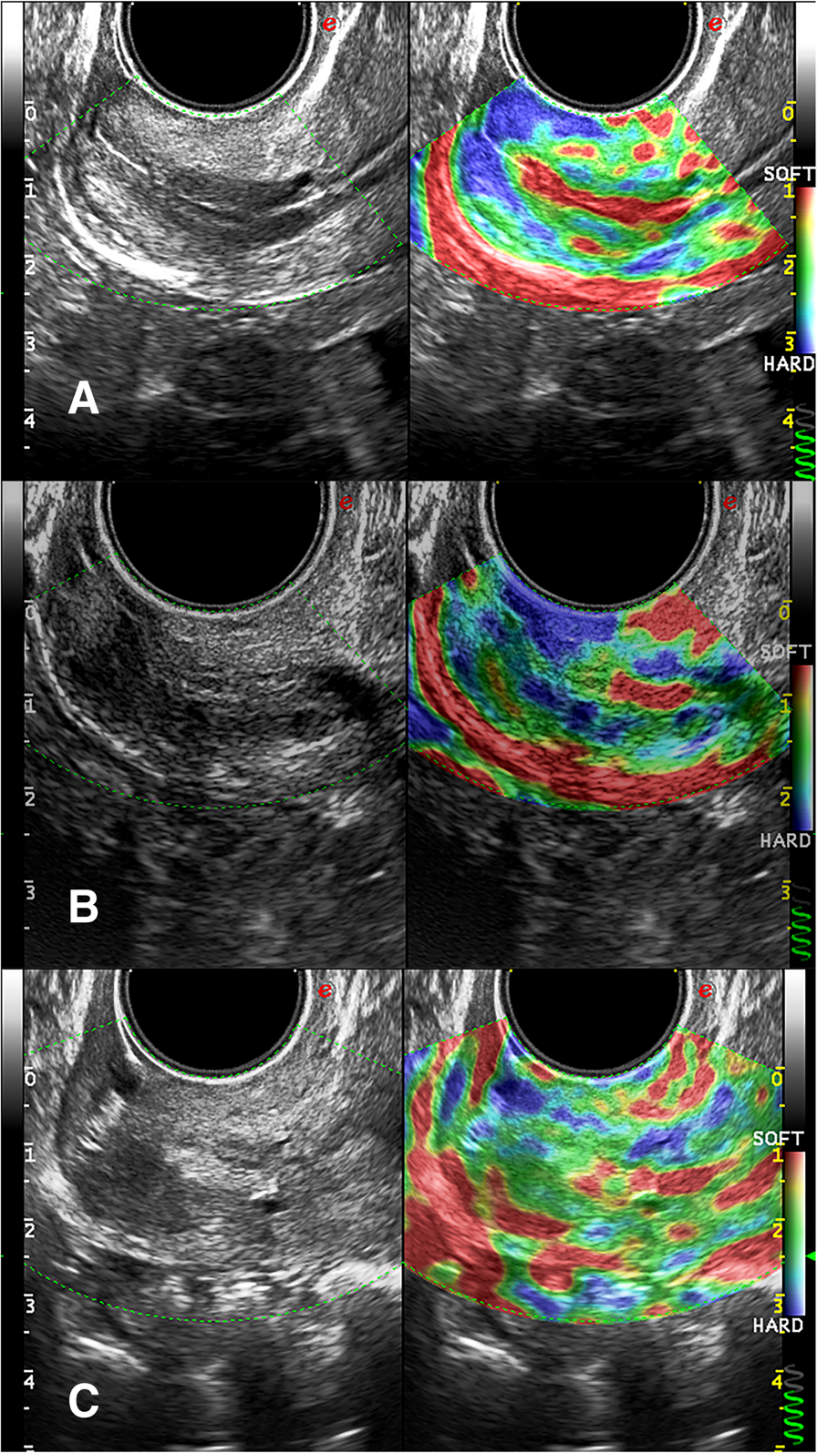
Comparison of elastographic evaluation in a case with easy, difficult and impossible embryo transfer. **a** Cervical channel appears *red*, while the surrounding tissue comprehensive of the six regions of interest (ROI) appears uniformly green with *rare blue* spots. Embryo Transfer (ET) resulted to be easy; **b** Cervical canal is not uniformly defined, and surrounding tissue shows a dishomogeneous *color pattern*. ET resulted to be difficult; **c** Cervical canal appears to be partially obstructed by a hyperechoic tissue in *grey scale* image, while elastographic image demonstrates the presence of diffuse tissue dishomogeneity. ET was not feasible, and we had to proceed with a transmiometrial ET

The ET was easily performed in 99 out of 154 patients (64.2%) and was difficult in 54 patients (35%). The ET resulted impossible in one case (0.65%) due to severe cervical stenosis, and therefore transmiometral embryo transfer was performed. Since routine ET was not feasible, this patient was excluded by the study. Cervical elastography revealed a very high DI value in this case (DI = 2.2, Fig. 2).

Table 1 displays the results obtained by the patients as stratified according to their ET pattern. Patients with easy and difficult transfer were comparable for age and ovarian reserve markers. On the other hand, the DI values obtained by the elastographic comparison of the six ROI resulted significantly higher in patients with difficult ET compared to patients with easy ET. No difference was seen in pregnancy rate among groups; yet the present study was not designed nor powered to detect this difference.

**Table 1:** Comparison of clinical parameters in patients stratified according to the ease of the embryo transfer (ET)

Parameter	Group 1 (easy ET)	Group 2 (difficult ET)	*p*
Age years	36.3 (0.93) [26–47]	36.7 (1.11) [29–44]	0.668
Basal FSH mIU/ml	7.2 (0.55) [2.2–19]	6.94 (0.71) [1.69–17]	0.683
Basal LH mIU/ml	5.47 (0.45) [0.8–17.3]	5.31 (0.69) [1.08–14]	0.434
Basal Estradiol pg/ml	57.1 (8.7) [9.7–329]	51.5 (7.7) [5.7–181]	0.713
AMH pmol/L	3.1 (8.79 [0.1–13]	2.9 (0.75) [0.01–11]	0.280
A/B Dishomogeneity index (DI)	0.25 (0.03) [0–1.3]	0.52 (0.06) [0.1–1.23]	0.007
C/D DI	0.17 (0.02) [0–0.52]	0.5 (0.06) [0.02–1.33]	0.001
E/F DI	0.22 (0.03) [0.02–0.9]	0.5 (0.1) [0–2.4]	0.004
Pregnancy rate	31/99 (31%)	20/54 (37%)	0.47^a^

Data are displayed as mean with 95% CI in parentheses and range in square brackets, unless otherwise specified. Comparison among groups were computed using two-tailed Mann Whitney U test or ^a^ two-tailed Z score for two population proportions

We then evaluated the predictive ability of the DI values on the ET ease by binary logistic regression model. Table 2 displays the comparison of the results obtained by three different models built with the individual evaluation of the DI values as obtained by the comparison of the stiffness of 6 different ROI (A/B, C/D, E/F) two by two. All models were statistically significant: among others, the model built with the C/D DI value, obtained from the evaluation of the cervical medial lips, correctly classified 86.9% of patients, with a sensitivity of 81.4% and a specificity of 89.9%, positive likelihood ratio (LR) 8.07 and negative LR of 0.21. The two other models obtained higher specificity but lower sensitivity and diagnostic accuracy (Table 2).

**Table 2:** Binary logistic regression analysis of the predictive ability of three distinct dishomogeneity index (DI) values as provided by cervical elastography in discriminating the cases with easy or difficult embryo transfer

Parameter	A/B DI	C/D DI	E/F DI
Chi quare (*p* value)	49.672 (*p* < 0.0001)	99.865 (*p* < 0.0001)	44.219 (*p* < 0.0001)
Diagnostic accuracy %	79.7	86.9	78.4
Sensitivity %	57.41	81.48%	51.8
Specificity %	91.92	89.9%	92.93
Positive LR	7.1	8.07	7.33
Negative LR	0.46	0.21	0.52
PPV %	79.4	81.48	80
NPV %	79.8	89.9	77.9

Finally, the predictive accuracy of the independent variables was quantified with AUC estimates derived from ROC curve. The AUC of the three models built with A/B, C/D and E/F DI value were 0.84, 0.92 and 0.77 respectively. The C/D DI cut-off value of 0.29 predicted the difficulty of ET with a sensitivity of 88.9% and a specificity of 85%.

## Discussion

The results of the present study suggest that cervical elastography could represent a novel diagnostic tool for the prediction of embryo transfer ease. Elastographic comparison of tissue deformation degree (strain value) between two opposite ROI (strain ratio) is supposed to provide the degree of tissue homogeneity, according to previous studies [11]. As strain ratio value of 1 accounts for tissue homogeneity, we calculated 1-strain ratio/strain ratio-1 values and expressed them as dishomogeneity index (DI). Due to the lack of similar studies in infertile women, we had no reference zones in cervix to choose for elastographic evaluation: therefore we decided to examine six opposite zones of cervix separated by the cervical canal, located near to the external uterine orifice (A and B), in the median zone (C and D) and near the internal uterine orifice (E and F) respectively, as we were expecting that cervical pathologies that could impact on the ET ease would affect the zones located near the cervical canal..

We found that women with difficult ET had significantly higher DI values compared to those with easy ET in all cervical zones under examination. Among others, the median zone DI was found to be the best independent predictor of ET ease compared to the zones located near the external and internal uterine orifice, as it correctly classified 86,9% of cases and provided the highest sensitivity value compared to the other zones (81.4% vs. 57.4 and 51.8%). This finding may be explained by the different orientation of the fibrous connective tissue that composes the uterine cervix and that largely contributes to its mechanical properties. X-ray diffraction studies have revealed three zones of collagen within the cervix: adjacent to the canal and in the outermost zone the fibrils are oriented predominately longitudinally, i.e. parallel to the canal, while in the middle zone they have a preferred orientation in a circumferential direction [17]. On the other hand, optical coherence tomography has shown that fiber orientation may vary in some physiological circumstances: in non pregnant women the cervical samples fibers are more regular compared to those in pregnant women [18]. In pathological circumstances such as in women with pyometra, the fiber orientation pattern may be disrupted because of collagen degradation and connective tissue remodeling [19], and modification in glycosaminoglycans composition as found during pregnancy or induced by infection may lead to alteration in cervical tensile strength [20]. We hypothesize that the pathologies that may alter the cervical canal patency may more significantly disrupt the middle zone fiber orientation due to their circumferential direction. Pathological studies are, however, needed to support this hypothesis.

It has been demonstrated that progesterone modulates collagen structure and estrogens modulated the elastic fiber structure of the cervix, therefore their increase during ovarian stimulation could affect the cervical tissue stiffness. Actually, this effect seems to become relevant only during pregnancy, when the structural reorganization of collagen and elastic fibers leads to changes in cervical tissue stiffness that may be crucial for cervical ripening, while in non pregnant mice the degree of cervical stiffness remained unmodified during the whole menstrual cycle. [21]. Nevertheless, in order to minimize the possible interference of increasing estradiol and/or progesterone levels on cervical tissue stiffness during ovarian stimulation, patients cervical tissue evaluation by ultrasound elastography was performed on day 2, before the start of the gonadotropin administration.

A limitation of the present study is that we couldn’t compare the results of the index diagnostic test to a reference gold standard, since no standard test have been validated in this field [8]. We tried to overcome this limitation by assigning the ET procedure to an expert operator, who was blinded to the results of cervical elastography, and by using a unique catheter, the Edwards Wallace, aiming at avoiding misinterpretation of ET ease possibly occurring with the use of stiffer catheters. We also tried to prevent selection bias by enrolling only patients at their first IVF attempt with no history of IUI, so that the ET ease couldn’t be foreseen. We are also aware that this test would require a further validation in a second independent group of patients, possibly by an independent group of researchers.

## Conclusions

In conclusion, the present study suggests that the evaluation of cervical tissue dishomogeneity by ultrasound elastography may predict the embryo transfer ease in infertile women candidates to IVF. These results could allow the identification of a subgroup of patients who could benefit from the use of cervical dilation prior to ET in order to improve their IVF outcome.

## Abbreviations

DI: Dishomogeneity index
ET: Embryo transfer
ICSI: Intracytoplasmic sperm injection
IVF: In vitro fertilization
ROI: Region(s) of interest
